# How family physicians in Singapore recognise complexity during consultations: a qualitative study

**DOI:** 10.1186/s12875-024-02368-7

**Published:** 2024-04-25

**Authors:** Jing Sheng Quek, Eng Sing Lee, Lian Leng Low, Sabrina Kay Wye Wong

**Affiliations:** 1 National Healthcare Group Polyclinics, 3 Fusionopolis Link, Nexus@one-north, South Tower, # 05-10, Singapore, 138543 Singapore; 2 Lee Kong Chian School of Medicine, Nanyang Technological University Singapore, Headquarters & Clinical Sciences Building, 11 Mandalay Road, Level 18, Singapore, 308232 Singapore; 3 Department of Family Medicine and Continuing Care, Singapore General Hospital, Academia, 20 College Road, Singapore, 169856 Singapore; 4 Outram Community Hospital, 10 Hospital Boulevard, Singapore, 168582 Singapore

**Keywords:** General practice, Primary health care, Complex care, Multimorbidity

## Abstract

**Background:**

The prevalence of persons with complex needs in Singapore is rising. Poor understanding of what constitutes complexity impedes the identification of care gaps and development of interventions to improve care for these individuals. We aim to identify the characteristics contributing to complexity in primary care, from the Family Physicians’ (FP) perspectives.

**Methods:**

Focus group discussions (FGDs) were conducted from January to September 2021 with experienced FPs across 14 study sites, employing a qualitative descriptive approach based on a complexity framework. Data were coded independently and categorised using thematic analysis by two independent investigators.

**Results:**

Five FGDs were conducted with 18 FPs aged 32 to 57 years old working in different primary care settings, with a mean of 13.5 years of primary care experience. Participants emphasised the need for a unified definition of complexity. Complexity is characterised by the presence of issues spanning across two or more domains (medical, psychological, social or behavioural) that adversely impact medical care and outcomes. Persons with complex needs contrast with persons with medically difficult issues. Medical domain issues include the number of active medical problems, poor chronic disease control, treatment interactions, ill-defined symptoms, management of end-of-life conditions and functional impairment. Psychological domain issues include the presence of mental health conditions or cognitive impairment. Social domain issues include the lack of social support, competing social responsibilities and financial issues, while behavioural domain issues include a lack of trust in healthcare workers, fixed health beliefs and poor health literacy.

**Conclusion:**

Recognising the medical, psychological, social and behavioural factors that contribute to complexity aids in discerning the diverse needs of individuals with complex needs. This underscores the need for additional support in these pertinent areas.

**Supplementary Information:**

The online version contains supplementary material available at 10.1186/s12875-024-02368-7.

## Background

Singapore’s population is aging rapidly. The proportion of those 65 years old and above increased from 9% in 2010, to 15.2% in 2020, and will reach 25% in 2030 [1]. With increasing age, the risk of multimorbidity rises [2, 3]. Against this backdrop, there exists a subset of individuals whose care needs are particularly complex. Increased complexity has been associated with higher healthcare resource use, longer consultation times, and suboptimal clinical outcomes [4–6]. It is acknowledged that management of complexity requires additional resources. It is only with accurate identification of persons with complex needs that we can match them with the requisite healthcare and social resources [7, 8]. However, population-wide assessment of complexity and its associated care requirements is currently suboptimal [7].

Many definitions of complexity in primary care have been proposed. However, the lack of a standardised definition of complexity prevents care needs from being categorised uniformly, and complicates research efforts aimed at understanding and addressing complexity [9]. Some studies equate complexity with multimorbidity, but most acknowledge that complexity goes beyond numerical disease count and is multi-dimensional [10, 11]. Other studies identify complexity through higher healthcare resource use, but this definition excludes complexity that is associated with treatment non-adherence or refusal [12]. Several conceptual frameworks have defined complexity as the interplay between medical and non-medical factors that influence health outcomes [12–15]. These frameworks highlight the importance of cultural and community factors, physical environments and local healthcare systems [12, 16]. Factors that contribute to complexity are likely to differ among countries due to differences in healthcare systems, culture and social factors, underscoring the importance of local evidence in the study of complexity [16]. However, the current dearth of studies on complexity in Singapore and Asia presents an important barrier to identifying persons with complex care needs and impedes the development of strategies to improve care provision.

In studying complexity, it is suggested that mixed methods approaches may better capture its uncertain nature over conventional randomised controlled trials predicated on linearity and predictability [17]. It is likely that a physician’s perception of complexity will be seen through the lens of their specialty and the profiles of patients that they care for [18, 19]. Primary care physicians (PCPs) are the first line of care for many persons with complex needs, managing them in the context of their community and family, and coordinating care with hospitalists and community services [20]. Therefore, understanding PCPs’ perspectives on what defines complexity will facilitate the identification of gaps in current care, and the provision of resources to better support these individuals [18, 19].

Primary care in Singapore is delivered by publicly-funded polyclinics and privately-funded General Practitioner (GP) clinics. Polyclinics are ‘one-stop’ facilities that house physicians, nurses and allied healthcare professionals (AHPs), with basic laboratory and radiological capabilities [21]. GP clinics, on the other hand, vary widely from individually-owned clinics to practices run by medical groups with in-house diagnostic facilities [22]. Individuals with chronic conditions tend to seek care at polyclinics due to higher subsidies and accessibility to services [23]. The 2014 Primary Care Survey showed that 52% of polyclinic attendances were for chronic disease management, while only 20% of attendances at GP clinics were for chronic conditions [24].

To better understand the issues faced with provision of care for persons with complex needs in the unique context of the Singapore primary healthcare system, this study sought to identify characteristics contributing to complexity from the perspectives of Family Physicians (FPs) working in various practice settings in Singapore.

## Methods

### Design

We conducted focus group discussions (FGDs) amongst experienced FPs in Singapore, employing a qualitative descriptive approach [25], that is based on a Complexity Framework proposed by Schaink et al [12]. The Complexity Framework describes a high-level framework to guide research development and approach to complexity, comprising five domains, namely: (i) medical/physical health, (ii) mental health, (iii) social capital, (iv) health and social experiences, and (v) demographics.

Ethics approval was obtained from National Healthcare Group Domain Specific Review Board (2019/00524).

### Sampling and recruitment

In Singapore, FPs are doctors with postgraduate Family Medicine (FM) qualifications (either a diploma or masters in FM) and adequate clinical experience, and are accredited with the national Family Physicians Accreditation Board [26]. The perception of complexity is likely to be influenced by clinical experience and training [27]. However, this study’s primary aim was to better understand complexity in the context of the existing healthcare system. Therefore, only FPs with minimally five years of primary care experience and who were in active clinical practice were included, to minimise the influence of inadequate experience that is related to a lack of post-graduate FM training.

Participants worked in one of the three polyclinic groups in Singapore (National Healthcare Group Polyclinics, National University Polyclinics and SingHealth Polyclinics) or in a privately-funded GP clinic. They were invited via e-mail and informed consent was obtained prior to the FGD. Respondents who agreed to participate in the study were sent a password-protected informed consent form, which they completed and sent back to the study team before the commencement of the FGD. Purposive sampling was conducted to obtain variation in place of practice and gender within each focus group. Recruitment ceased when the study team determined that data saturation was met and no new complexity domains were identified.

### Focus group discussion

FGDs, each lasting 60 to 120 min, were conducted in English over a videoconference platform between January 2021 and September 2021. Strict cybersecurity measures were undertaken, including the use of an enterprise account (with enhanced security features in accordance with institutional information technology standards which was also used in clinical settings for tele-consultations), the sending of private meeting details to participants individually, the creation of waiting rooms before admission by study team members after identity verification and the disabling of private chat and file transfer functions. Each FGD was facilitated by two out of three researchers (two with PhD and one with master of clinical investigation), all of whom were trained in qualitative methods and in active primary care practice as Senior Consultant Family Physicians. All researchers shared with interviewees their interests in complexity research. A research assistant was also present for administrative support.

A semi-structured interview guide was developed, based on the complexity framework developed by Schaink et al. and literature review [12–16]. We pre-tested the guide on 5 FPs, and made iterative changes after pre-testing and informed by findings of the FGDs. Each participant prepared two examples of persons with complex needs they had encountered in the preceding month and the interviews commenced with participants sharing about the complex needs they had encountered in their practice. The guide also included probing questions on domains that were associated with complexity. Interviewers took individual field notes, and the interviews were audio-recorded digitally, transcribed verbatim and verified by a study team member.

### Data analysis

Each transcript was independently coded by two researchers. Thematic analysis was performed. Issues contributing to complexity were identified using initial open coding. The coding scheme was refined iteratively by moving between coding and revising the codes, until all team members came to an agreement on the coding scheme. Individual codes were subsequently mapped to one of the five dimensions of the Complexity Framework [12]. Dimensions were removed, combined, created and renamed into themes known as domains. NVivo 12 (QSR International, 2018) was used to organise the data. Data saturation was reached after conducting five FGDs involving 18 FPs. This study was reported according to the Consolidated Criteria for Reporting Qualitative research (COREQ) checklist [28]. Repeat interviews were not carried out and transcripts were not returned to participants for comments or checking.

## Results

### Sociodemographic characteristics

The response rate was 37.5%. Most respondents did not state any reason for refusal, but those who did cited clash with schedule as reason for non-participation. Eighteen participants aged 32 to 57 years old were interviewed. Fourteen participants from 10 different polyclinics and 4 participants from 4 different GP clinics took part. Participants had a mean of 13.5 years of primary care experience. Fifteen of the participants spent at least half of their working hours on clinical work (see Table 1).

**Table 1:** Characteristics of participants

Characteristics	Number (%)
**Gender**	
Male	12 (67%)
Female	6 (33%)
**Age group at interview (years)**	
30–39	8 (44%)
40–49	5 (28%)
50–59	5 (28%)
**Type and Place of Practice**	
Private, General Practice (GP) Clinic	4 (22%)
Public, National Healthcare Group Polyclinics (NHGP)	7 (39%)
Public, National University Polyclinics (NUP)	3 (17%)
Public, SingHealth Polyclinics (SHP)	4 (22%)
**Duration of primary care practice (years)**	
5–10	9 (50%)
More than 10	9 (50%)
**Highest postgraduate Family Medicine (FM) training qualification**	
Graduate Diploma in Family Medicine [GDFM]	1 (6%)
Master of Medicine in Family Medicine [MMed(FM)]	6 (33%)
Fellowship of the College of Family Physicians Singapore [FCFP(S)]	11 (61%)
**Mean clinical Full Time Equivalent (FTE) in the past year**	
0.10-0. 50	3 (17%)
0.51–0.75	8 (44%)
0.76-1.00	7 (39%)

### Importance of defining complexity

Participants emphasised the need for an aligned definition of complexity as a basis for guidelines development, training, resource allocation and facilitation of research.

We need to know what is the definition of complexity first before we can then say what to do. If I’m talking about biomedical complexity, then definitely more training, practice, exposure to similar issue[s]. But if we […]look at complexity because of a lot of psychosocial issues[…], we will think about social determinants of health. And that’s where a lot of the policy and environment [issues] will then affect how complexity will play out. (P11)

### Complex versus medically difficult

Among persons who required more clinical effort, two distinct categories were described: the person with complex needs and the person with medically difficult issues. Many FPs did not consider a person with predominantly medically difficult issues as having complex issues.

The difficult patient more pertains to a medical condition that is difficult to manage[…], but this patient may not be a complex patient. (P10)

The person with complex needs, on the other hand, had issues across multiple domains (medical, psychological, social and behavioural) that impacted medical care and outcomes (see Table 2). Participants stated that these individuals required longer consultation time and needed a multi-disciplinary team approach.

To me, complexity is the multiple domains that it cuts across that even within whatever expertise you have in the area, it still requires a lot more effort to manage them. (P02)

FPs felt that for persons with medically difficult issues, outcomes were still achievable. However, for persons with complex needs, there was no straightforward way to achieve the desired outcomes.

I would find someone to be complex if there is no straightforward way to achieve the outcome… For example, a patient […with] poorly-controlled [diabetes], […]if it’s just up to me, titrating the medication, […]the patient will […]just be medically difficult and I can still achieve the outcome. But if the patient has very poorly-controlled diabetes and there are psychosocial issues, financial difficulties, then there is no straightforward way for me, […]to achieve the outcome that I want for this patient, and I may have to pull in additional resources, whether it’s community health partners or perhaps, referral to a secondary institution, then it becomes complex. (P01)

The need to involve community social agencies differentiated persons with complex needs from those who were medically difficult. Some FPs recognised that in order to improve the individual’s medical issue, the non-medical issue had to be addressed in tandem.

There is [an] interlink between his social circumstances and his medical problems. You can’t just tackle the medical problems alone without looking into his social circumstances. (P01)

**Table 2:** Characteristics of complex persons

Domain	Characteristics
Medical	Number of active medical problems
	Poor chronic disease control
	Treatment interactions
	Unclear diagnoses or ill-defined symptoms
	End-of-life care
	Functional impairment
Psychological	Mental health conditions
	Cognitive impairment
Social	Lack of social support
	Competing social responsibilities
	Financial issues
Behavioural/ Literary	Lack of trust in healthcare workers
	Fixed health beliefs
	Poor health literacy

### Contributors to complexity

#### Contributors to complexity – medical domain

In all the examples of persons with complex needs described by the participants, at least one active medical problem was present. As described by a participant, “it’s not the number of diagnoses, but the number of active medical problems that require ongoing attention” (P15) that contributed to complexity.

Poor control of chronic diseases was the most commonly-identified factor. This frequently results in acute symptoms, multiple healthcare visits, and increases the risk for complications.

And the difficulty in controlling, I think is extremely important[…] So there could be acute or chronic exacerbations of [a chronic disease], there could be just chronically persistent, uncontrolled [chronic disease]. (P14)

Treatment interactions, where the treatment of one condition affects the outcomes of another, contribute to complexity. Polypharmacy tends to aggravate the issue. Balancing the benefits of a treatment and its adverse effects increases complexity.

You want to treat this, but […] you end up with side effects of the treatment or triggering some other conditions. (P18)

Individuals with ill-defined or psychosomatic symptoms causing unclear diagnosis contribute to complexity, because they would require multiple consultations and specialist referrals.

It’s so undifferentiated that I don’t know whether to send to ENT, neuro[logy] or […]every single speciality. […P]atients […]go one big round after specialist[s] consult[s], they come back to the GP for us to solve their problem, with the various input from the specialists. And then you have to make sense of the data and to hold the hands of these patients. (P13)

Symptomatic end-stage conditions contribute to complexity. These symptoms require frequent clinic visits with medication adjustments. There is also a lack of experience in managing such individuals.

[…O]ne patient that came here for dry gangrene on the feet […]didn’t want [surgery], but the son literally begging the nurse to just do something because she’s always in pain. I started morphine. With whatever palliative knowledge I had, I tried to organise [hospice services], even though [s]he refused to […]see palliative. So […]every day I […]see her, titrate the morphine, call my palliative friends… (P05).

Frailty with functional impairment, especially in the presence of frequent falls, contributes to complexity. There are usually multiple reasons for falls and preventing further falls is challenging, particularly when social issues are present.

[…T]his 60 year old Indian lady was [referred] just for a simple [removal of stitches] for her left eyebrow laceration. On further history-taking you realised she’s had about 6 serious falls in the last 2 years[…]. She has many predisposing factors, so that’s why she kept falling. […]We got in the social worker, […]went for a home visit and then [uncovered] a lot of other social issues, so eventually had to get […]home care, […]quite a lot of help eventually, but all these took […]almost 2 years. (P08)

#### Contributors to complexity – psychological domain

Mental health conditions were highlighted as an important contributor to complexity, impacting diagnosis, management, and disease outcomes. Commonly-identified mental health issues include depression, anxiety disorders, schizophrenia, acute stress reactions, bereavement and personality disorders.

Persons with co-existing mental health issues may present atypically, increasing diagnostic complexity.

Patients with anxiety or anxious personality trait tend to really jot down every single detail and the more information they give you sometimes, you really got to learn how to sit back and piece them together. (P13)

Individuals with mental health issues may display behaviours that make their management challenging, for example by being dependent on others, or refusing treatment. Consequently, their chronic disease outcomes are adversely affected.

[I]t’s very complex, that she’s not really having pathological grief[…], but a lot of change in her lifestyle without her husband because she was quite dependent on the husband, actually affect her mood… then it will affect her diabetes control, affect her lifestyle, affect her compliance to medicines… (P11).

Individuals with cognitive impairment (e.g., dementia or intellectual disability) tend to have poorer decision-making capacity, health literacy and self-care behaviours, contributing to complexity. FPs also described difficulties with involving caregivers in caring for them.

[…]We have to corroborate the history with the daughter, but […]the daughter does not stay with her and only sees her once a week[…]. There are a lot of issues with regards to the reliability of the history, especially the dietary part. The pill box is supposed to resolve most of the problems, […]but we do not know if it’s being taken at the right time, whether she drops any medicine. (P15)

Another participant shared about the impact of an individual’s intellectual disability on his present and future care.

This person has […]intellectual disability and he lives alone. And because of his intellectual disability, he has very poor insight into his chronic conditions, […causing] non-compliance to diabetes treatment and lifestyle advice. Decision making is a challenge for him, for current as well as for future. (P10)

#### Contributors to complexity – social and behavioural issues

A lack of social support, presence of behavioural issues or poor health literacy are contributors to complexity.

For persons with psychological, cognitive or functional issues, lack of functional support contributes to complexity. Functional support includes assistance with activities of daily living (e.g. bathing, travelling to medical appointments) or self-management of chronic diseases (e.g. medication adherence, health parameters monitoring).

Social isolation comes together with the lack of care, so the patient is on his or her own. There’s no support, be it medically, like you need somebody to administer medication, need somebody to make sure that medication is well taken, somebody […to] buy the meals. (P05)

Lack of emotional support, especially in persons with chronic conditions, impacts on self-care behaviours, increasing complexity.

And you lack emotional support. You are alone. You don’t feel like there is a need to take care of myself. […]“Better that I just die off faster, not be a burden.” Maybe don’t have somebody to encourage to say, “Hey you are doing good, you can work on it.” (P05).

Presence of social issues also contributes to complexity because these individuals’ competing social responsibilities are prioritised over their health and self-care behaviours.

They also have other responsibilities. They themselves are patients, yet they are caregivers to elderly parents, a spouse who is also not well. So […]they might not think of their condition as important enough[…]. They actually up-prioritise the needs of their loved ones who are not well, and then don’t do much about their own condition because they have multiple social roles. (P06)

Financial issues compound complexity by contributing to non-adherence to medical appointments and/or medications, and could also lead to less social support.

[…T]he patient may skip medications and not be compliant intentionally to save cost. This is assuming that they are still seeing [the doctor]. In a higher social economic status family, there could be a helper […]to look after the […]dependent patient. So that could also be an indirect implication of the social economic [status]. (P15)

Two behavioural traits reportedly increase complexity. Firstly, a lack of trust in the healthcare system or team affects the individual’s adherence to medical recommendations.

Somebody who is very mistrusting or needs a lot of convincing before they agree to [or] adhere to any form of treatment. (P03)

Secondly, persons with fixed health ideas or poor insight into their conditions are complex to manage.

Some patients have fatalistic attitudes, some patients are indifferent, some patients have no insight. […T]hese are […]very tough because whatever you say, they either don’t get it, or they don’t really care. […T]hey come to you, they take their medication, but whether it’s well controlled or not, they are not very important. Either they don’t care, or they say, “Aiyah, I’m going to die already, so it’s ok. You know, I’m so old.” (P06).

Finally, persons with poor health literacy are unable to make appropriate health choices, and this increases the complexity of their care.

Her own underlying health literacies and her own abilities – I think that’s what added to the complexity of the case. […Y]ou can be a multimorbid patient, but if you’re health literate and you are motivated, it will not appear to be a complex case, [be]cause things are under control. (P02)

## Discussion

Our study found that FPs concurred that complexity was multidimensional, and spanned medical, psychological, social, and behavioural domains, adversely impacting medical care and outcomes. Participants also described a distinct group of persons with medically difficult issues who also require more clinical effort, however, issues are mainly limited to the medical domain.

Our study agreed with previous research regarding the multi-dimensionality of complexity [12, 13, 27]. In addition to medically challenging issues, complex needs included psychological, social and behavioural issues impacting on medical care and outcomes. This echoed existing literature that highlighted *interconnectedness* as a distinct element that differentiated complex from complicated [9], implying that no one domain was more important than another, and that the management of complexity necessitated addressing multiple issues simultaneously. This suggests that multi-disciplinary teams are required in primary care settings, with nurses and AHPs addressing mental health, behavioural and health literacy issues, and supporting the provision of end-of life care alongside FPs. In addition, the integration of primary care with community-based social services would also support the care of individuals facing complex social issues.

While the importance of defining complexity in primary care was recognised, its definition was not straightforward. For example, we found that active medical issues, rather than the total number of diagnoses contributed to complexity [10, 15]. The current definition of complexity used by the Singapore Ministry of Health for reimbursement focuses on chronic disease count, and may be an inaccurate reflection of complexity [29]. However, many of the factors identified, such as treatment interactions, social capacity, health literacy and health beliefs, were currently not coded in electronic medical records, impeding system-wide identification of complexity. The ability to identify complexity from electronic data sources will enable a more population-based approach to address complexity through resource allocation and reimbursement. However, further study will be required to understand the impact and utility of identifying these additional factors in supporting care of persons with complex needs.

There appeared to be differences in how physicians recognise complexity according to their sites of practice. For example, participants who brought up ambiguous diagnosis as contributors to medical domain of complexity mainly practised in private GP clinics. While this was not our primary research objective, further exploration of how specific practice contexts affected the perception of complexity could be undertaken in future research.

Poor trust in the healthcare team and strong ideas/preferences were identified as contributing to complexity, similar to findings of other studies [8, 13, 30]. Care continuity and strong patient-provider relationships may have greater importance for persons with complex needs. Existing studies have shown that care continuity is associated with reduced hospital admissions, and further studies could determine if the impact of care continuity or patient-provider relationship is even greater for people with complex needs [31, 32].

Our study findings emphasized the heterogeneous nature of complexity, echoing previous research that complexity exists on a spectrum [5]. Identification of multiple complexity factors provided a more comprehensive understanding of the diverse ways that complexity could present. However, translation of these findings into a comprehensive screening tool would be challenging for implementation due to the large number of factors present. Tools such as the INTERMED have been developed but its clinical use has been largely limited to research settings [33]. Future studies could consider analysing clustering characteristics in complexity factors, which may lend further insight into the relationship between various complexity domains, and potentially simplify identification of complex needs.

## Limitations and strengths

The findings of our study should be interpreted in the context of its limitations. Non-FP PCPs were not included in this study and issues with complexity that may be related to inadequate FM training would not have been explored. It is likely that gaps remain in FM training in relation to managing complexity, and further studies comparing perceptions of FM residents, non-FP PCPs and FPs would provide further insight. Nurses, AHPs and ancillary staff, who also provide care for persons with complex needs, may have a different perception towards complexity, and they were not included in this study too. Future studies incorporating their perspectives may provide further insights on multi-disciplinary care requirements.

Our study identified key domains of complexity that are encountered by FPs working in public and private primary care clinics in Singapore, adding to the findings of a recently published local study focusing on the challenges and enablers faced by private GPs in the management of persons with complex needs [34]. The descriptive qualitative approach of our study allowed for identification of the domains based on the real-world experiences of FPs from various places of practice, and lays the foundation for further research into this area.

## Conclusion

Our study found that persons with complex needs have issues that span multiple domains, including medical, psychological, social and behavioural. They were distinctly different from persons with medically difficult issues. Recognising the factors that contribute to complexity aids in discerning the diverse needs of individuals with complex needs. This underscores the need for additional support in these pertinent areas.

### Electronic supplementary material

Below is the link to the electronic supplementary material.

Supplementary Material 1

Supplementary Material 2

## Data Availability

The datasets used and/or analysed during the current study are available from the corresponding author on reasonable request.
