# Supplementary material for: How family physicians in Singapore recognise complexity during consultations: a qualitative study
Source: BMC Prim Care. 2024 Apr 25;25:134. doi: 10.1186/s12875-024-02368-7 (PMC11044365; doi:10.1186/s12875-024-02368-7)
Supplement: Supplementary file 1 — Supplementary Material 1 [file 12875_2024_2368_MOESM1_ESM.docx]

**Appendix A**

Interview Guide

Thank you for participating in this focus group discussion. We are interested in finding out your views on the characteristics of complex patients and would like to encourage all participants to contribute to the discussions.

1. We had requested that you prepare 2 examples of complex patients that are seen in your practice. Could you describe one of patients you have prepared?
   - Probes
     - Who would like to start first?
     - Would you like to share your experience as well?
     - What do you think of this?
     - Would you like to add to what has been shared?
   - Why did you consider this patient complex?
     - Other there are reasons?
2. Do you think that ___ contributes to patient complexity? (ask about each of the domains below)
   - Domains
     - Medical conditions
     - Treatment regime
     - Socioeconomic factors
     - Mental health conditions
     - Behavioral traits
     - Patient preferences and priorities
     - Cultural factors
     - Acute life events
     - Personal capacity and resilience
   - Could you give an example of describe how (insert domain) contributes to patient complexity?
   - Are there other areas that you think might contribute to patient complexity?
3. What do you think helps with the care of complex patients?
4. What are some of the issues faced by your complex patients?

Are there issues related to the following:

- Use of healthcare services
- Hospitalisation or ED visits
- Clinical outcomes
- Psychological health

**Appendix B**

E-mail invitation template

**A synthesis of primary care physician identified characteristics of complex patients using focus groups: the PLEX study**

**Subject:** Invitation to participate in an interview study

Dear Dr ______,

We would like you to participate in this study titled” A synthesis of primary care physician identified characteristics of complex patients using focus groups: the PLEX study”

**Purpose of study**

We aim to understand primary care physician identified characteristics of complex patients through focus group discussions held in five Asia-Pacific cities

**Why you are invited to participate**

You are invited because of your experience in caring for patients as a Family Physician working in a primary care setting.

**Study Procedures**

If you take part in this study, you will participate in a focus group discussion over Zoom that will include 4-5 Family Physicians, lasting about 2 hours. Before attending the focus group discussion, you will be asked to prepare a patient summary of 2 complex patients you have encountered in your practice, to be submitted in a password protected document via e-mail. During the focus group discussion, you will be invited to share about your 2 complex patients. A Participant Information Questionnaire will also be sent to you, and the password protected questionnaire document can be submitted via e-mail. The focus group discussions will be audio-recorded only, and your identity will be kept confidential.

**Participation is voluntary**

Your participation in this study is voluntary. You may stop participating in this study at any time. Your decision not to take part in this study or to stop your participation will not affect your work appraisal. If you decide to stop taking part in this study, you should tell the Principal Investigator.

**Person to contact for more information**

If you have questions about this research study, you may contact the Principal Investigator, Dr Sabrina Wong Kay Wye at [sabrina_kw_wong@nhgp.com.sg](mailto:sabrina_kw_wong@nhgp.com.sg) or 6355 3000.
